# Online Adaptation of Community-Based Teaching and Assessment in Response to the COVID-19 Pandemic: Student Feedback and Challenges

**DOI:** 10.7759/cureus.92920

**Published:** 2025-09-22

**Authors:** Ravneet Kaur, Aftab Ahmad, Kiran Goswami

**Affiliations:** 1 Centre for Community Medicine, All India Institute of Medical Sciences, New Delhi, IND

**Keywords:** adaptation, assessment, community-based teaching, covid-19, medical students, online

## Abstract

Background: Community-based teaching (CBT) is a crucial component of medical education that develops a comprehensive understanding of health and disease. Due to the COVID-19 pandemic, online adaptation of CBT and its assessment was required to ensure the continuation of teaching while maintaining physical distancing. After the pandemic, the online and blended learning modes are being increasingly adopted in medical education. It is likely that a similar mode of teaching and assessment may continue in the near future. The students' feedback can help in improving the system for acceptability and usability. In this study, we have documented the process of conducting online CBT and its assessment for Urban Health Posting (UHP) in Community Medicine and the feedback from medical students regarding the online adaptation.

Methods: The existing activities under the UHP were converted into suitable online activities, and an online assessment was conducted. A total of 58 students from three consecutive batches posted for UHP participated in the study. Feedback was obtained by using structured Google Forms (Google LLC, Menlo Park, California, United States), having both closed- and open-ended questions. Data were analyzed using Microsoft Excel (Microsoft Corporation, Redmond, Washington, United States), and results were summarized as percentages. Thematic analysis was conducted for open-ended responses.

Results: The majority (n=49), i.e., 84.4%, of students were satisfied with the online adaptation of CBT. They found it convenient, time-efficient, and engaging. Lack of immersive experience was cited as a major limitation. Access to the internet and its cost were also identified as challenges. Twenty-nine (50%) students faced problems in the online assessment, viz., inadequate time, poor internet connectivity, and difficulty in navigation. The support provided during the assessment was appreciated.

Conclusion: The online adaptation was convenient and engaging, but lacked field experience. Suggestions to improve online assessment included a user-friendly layout, adequate time, detailed feedback, and anti-cheating measures.

## Introduction

Community-based teaching (CBT) is a crucial component of medical education. Under this program, teaching and training are carried out in the community, beyond the confines of medical college and teaching hospital, to foster a comprehensive understanding of health and disease. The learners engage actively with the community and patients in a real-life context and understand the influence of psychosocial, cultural, and environmental factors on health [1,2].

The COVID-19 pandemic posed unprecedented challenges for medical education. The imposition of a complete lockdown, including closures of medical institutions, prompted the medical teachers to explore innovative ways to maintain teaching-learning. Use of technology in the form of e-learning proved promising in ensuring the continuation of teaching while maintaining physical distancing [3]. However, most of the e-learning occurred in the form of online lectures which were largely didactic and catered to the lower domains of Miller's pyramid, i.e., acquisition of knowledge. The CBT aims to develop higher skills in the cognitive, affective, and psychomotor domains, such as the application of knowledge and critical thinking [4]. An adaptation of CBT to suitable and feasible online components was the need of the hour. Hence, we developed a framework for the Urban Health Posting (UHP) component of CBT in online mode, including online assessment. We also gathered the feedback of medical students regarding CBT and online assessment.

Due to its unique advantages, the integration of technology in teaching and assessment is likely to persist in the near future. Systematic incorporation of students' feedback can play a crucial role in enhancing the system's acceptability, usability, and overall effectiveness.

In this paper, we share our experience of conducting CBT and its assessment in online mode during the COVID-19 lockdown period. We also document medical students' feedback regarding online CBT and its assessment.

Objectives

This study aimed to document the online adaptation of UHP during COVID-19 and to assess medical students' feedback on its effectiveness and challenges.

## Materials and methods

Study design

This was a cross-sectional study.

Study participants

The study participants were fifth-semester medical undergraduates who were posted in Community Medicine for their UHP. 

A total of 58 students (three consecutive batches posted for UHP) were included in the study. The sampling was purposive as all the students who appeared for the assessment and consented to participate in the study were included. 

Study duration

The study was conducted from July 16, 2020, to November 30, 2020.

Study settings

The study was conducted at the All India Institute of Medical Sciences, New Delhi, India. In other parts of the country, CBT is the responsibility of the Department of Community Medicine. The CBT had two components: urban and rural. The UHP was covered in the fifth semester, while the rural posting was covered in the seventh semester. This study pertains to the UHP component of the CBT program.

The Urban Health Program of the Department of Community Medicine provided community-based health services to the marginalized population of an urban resettlement colony in south-west Delhi. The program provided preventive, promotive, and curative health services and basic laboratory services to its population through an urban primary health centre. A team of doctors and health workers provided the clinical and community-based services. The program served as an essential platform for undergraduate education in Community Medicine. The students were posted under the program for 45 days to learn the competencies and skills related to Community Medicine for undergraduate medical students [5].

Activities under the UHP in pre-COVID-19 times

In the pre-COVID-19 times, the UHP was conducted in batches of 20 students each. The batch was posted for a period of 45 days, for four hours on each working day. The following activities were undertaken:

Briefings

These were interactive sessions aimed to orient the students about the functioning of the UHP and major teaching-learning activities to be held under the UHP. The briefings also provided an orientation and practical training on common clinical topics, such as common medical conditions, maternal and child health, immunization, documentation, etc., that the students need to be familiar with when they go into the field and the urban health clinic. Each session lasted for about 45 minutes. 

Clinico-Psycho-Social Case Review (CPSCR)

The students visited an urban resettlement colony and made home visits to gain an understanding of the medico-social and psychological aspects of illness. Core activities include patient history-taking, clinical examination, evaluation of the physical environment and Water, Sanitation, and Hygiene (WASH) practices, and exploration of psychosocial dynamics and family relationships. Mapping the web of causation and disease progression, prioritizing health needs from individual, family, and community perspectives, and identifying gaps in care and health-seeking behavior were the other activities under CPSCR. The exercise concluded with a student-led presentation that synthesizes case findings, diagnoses, and proposed clinical, dietary, social, and communication-based interventions.

Epidemiological Exercise

The students worked in teams and conducted a small-scale research activity in the community, with the aim to learn research methods and application of epidemiology and biostatistics. Under the guidance of the faculty, the students developed a research question and research objectives, drafted a research protocol, collected data using suitable tools, analyzed the data obtained, and presented the results.

Clinical Posting

The students visited the urban health centre, run by the Centre of Community Medicine. They were posted in the outpatient department (OPD), antenatal clinic (ANC), and immunization clinic of the urban health centre for four days each. This includes observing the interaction between the staff (doctors/pharmacists/laboratory technicians/health workers) and the patients. The students also learned the management of common ailments under the guidance of a senior resident doctor. 

Health System Visits

The students visited other public health facilities in the area, including the district hospital, dispensaries of the state government, Anganwadi centres, etc., with an objective of learning about the levels of healthcare, management of common ailments, and referral mechanisms. 

Health Education

The students learned the practical application of health education methods by conducting health talks in the community. They were supported by the faculty, residents, and community health workers.

Online adaptation of UHP activities

Due to the ongoing COVID-19 pandemic, all the activities under the UHP were converted to suitable and feasible online activities. This required considerable planning and innovation to ensure that the learning objectives, focusing on primary healthcare delivery, social determinants of health, and hands-on clinical exposure, could still be met despite the absence of in-person fieldwork.

The following activities were performed:

Review of Existing Literature

We conducted a review of published studies regarding online adaptation of clinical and CBT. Databases such as PubMed, Scopus, and Google Scholar were searched. The review highlighted innovative strategies adopted worldwide during the pandemic. It also provided an insight into challenges such as limited internet access, engagement issues, and maintaining the authenticity of community learning experiences, which guided the planning process.

Mapping of Available Resources

We undertook a systematic mapping of institutional and external resources that could support the online adaptation. This included the identification of existing Learning Management Systems (LMS) like Google Classroom (Google LLC, Menlo Park, California, United States) and Microsoft Teams (Microsoft Corporation, Redmond, Washington, United States), as well as video conferencing tools such as Zoom (Zoom Communications, Inc., San Jose, California, United States) and Google Meet (Google LLC, Menlo Park, California, United States). The institute had its own LMS called SARAL (Student Advanced Resources and Learning). The institute also provided access to an online platform (Jitsi Meet) to all its students and faculty to facilitate online teaching during the COVID-19 pandemic. It was decided to use the platforms provided by the institute. 

Discussion Among Faculty Members and Resident Doctors

It helped in contextualizing the literature review findings and resource mapping to address the specific needs and constraints in the given situation. Faculty members expressed their concerns regarding sustaining student engagement, replicating the experiential learning components of field postings, and ensuring fair evaluation of student performance in an online format. Resident doctors provided practical insights into feasible modifications. Consequently, it was decided to implement a blended pedagogical model that incorporated synchronous sessions, interactive assignments, and reflective exercises focused on urban health themes.

Based on these activities, a structured online teaching schedule was developed for the entire eight-week posting.

Briefings

Daily sessions were conducted via video conferencing platforms such as Google Meet or Zoom. These sessions typically ran from 10:00 AM to 1:00 PM and included live lectures, interactive discussions, and student-led presentations. Faculty members delivered interactive lectures on introduction to urban health, common medical conditions, CPSCR, and the structure of the health system in India. Students were encouraged to engage actively through chat functions, through verbal participation, and by presenting thematic reflections. Each session had dedicated time for questions, clinical case scenarios, and discussion of real-world applications.

Clinico-Psycho-Social Case Review (CPSCR)

The students were instructed to identify suitable cases within their own households. These cases were then presented in structured group sessions using PowerPoint slides. The students presented from their respective places (hostels or hometowns), while the faculty members (5-7) attended from their workplace. The faculty provided detailed feedback on the content, analysis, and communication skills. 

Epidemiological Exercise

The community research component was similarly adapted. In place of conducting a field-based survey, the students collaborated on an online research project titled "Assessment of Sleep Quality and Screen Time During the COVID-19 Lockdown Among Undergraduate Medical Students". Students were introduced to key concepts in epidemiological study design, ethics, questionnaire construction, and data handling. Once data collection was completed, subsequent online sessions focused on data cleaning, coding, and basic analysis using statistical software (Stata, StataCorp LLC, College Station, Texas, United States). Students were supported in creating tables and interpreting preliminary results.

Health System Visits

To address the lack of institutional field visits during the COVID-19 lockdown, a self-directed field-based assignment titled the "Nearby Health Facility" module was introduced. Students were asked to identify and visit two or more government health facilities in proximity to their place of residence, wherever feasible and safe under prevailing local guidelines. 

A standardized PowerPoint template was provided to guide data collection and ensure uniformity. Students recorded facility names, addresses, types, distance from home, and geo-tagged maps. They also documented the range of services offered (preventive, promotive, curative, and rehabilitative), nearby COVID-19 testing and admission facilities, and commonly available drugs and investigations, along with their indications, dosage, cost, and interpretation of results.

These presentations were made during scheduled online sessions and reviewed by the faculty. 

Table 1 shows the activities under UHP in the pre-COVID-19 period and their online adaptation during the COVID-19 period.

**Table 1:** Activities under UHP in the pre-COVID-19 period and online adaptation during the COVID-19 period MCQ: multiple-choice questions; OSPE: Objective Structured Practical Examination; CPSCR: Clinico-Psycho-Social Case Review; AWC: Anganwadi centre; OPD: outpatient department; ANC: antenatal clinic; SRs: senior residents; JRs: junior residents; MSSO: medical social service officer;  UCH: urban health centre; OSCE: Objective Structured Clinical Examination; UHP: Urban Health Posting

Pre-COVID-19			COVID-19 lockdown	
Activity	Method of teaching	Assessment	Adaptation of the activity	Assessment
Briefings for health topics	Face-to-face lectures	MCQ and OSPE	Interactive online lectures	Online MCQ-based test
CPSCR	Domiciliary visits and case work-up	Presentations to the faculty	Suitable cases within their own household were identified and prepared by the students	Online presentation in structured group sessions using PowerPoint slides
Epidemiological exercise	Community-based research project	Presentations to the faculty	A research project that included online data collection was carried out	Online presentations to the faculty
Health facility visits	Visits to health facilities such as AWC, district hospital, etc.	Submission of a written report	Nearby health facility visits, with a special focus on facilities for COVID-19 care	Online presentation
Clinical postings (OPD, immunization clinic, ANC)	Observation of the interaction between the staff (SRs/JRs/pharmacists/lab technicians/MSSO) and the patients attending the UHC. Work-up of clinical cases under the supervision of a senior resident	OSCE	Carried out at the time of the health facility visit	Items included in the online assessment
Health education	Health talks, role-plays in the urban field practice area	Feedback from the faculty, residents, and health workers	Community-based component was missed. The students developed health education material for the cases worked up for CPSCR	Review by the faculty members

Assessment framework

The activities were assessed on the basis of pre-defined criteria. At the end of their posting, the students were evaluated by an Objective Structured Practical Examination (OSPE) that was based on various aspects covered in the posting and the learnings from health facility visits. The assessment framework is shown in Table 2.

**Table 2:** Framework for the assessment of activities under the UHP *The end posting assessment was based on the topics covered during the briefings and the learnings from the health facility visits. In the pre-COVID-19 period, it was conducted in the form of an OSPE session, with different stations. During the COVID-19 period, it was conducted as a synchronous online session, in which an MCQ-based test was conducted. OSPE: Objective Structured Practical Examination; MCQ: multiple-choice questions; CPSCR: Clinico-Psycho-Social Case Review; UHP: Urban Health Posting

Activity	Maximum marks	Criteria for assessment
*End posting examination	15	Performance in OSPE (pre-COVID-19) and online MCQ-based test (during the COVID-19 lockdown)
Epidemiological exercise	15	Contributions to the group research project, including protocol development, data collection using online tools, and participation in data analysis and presentation
CPSCR	7.5	Completeness of history, clinical and psychosocial insights, web of causation, formulation of interventions, and clarity of presentation
Health education	7.5	Relevance, appropriateness, clarity, and scientific accuracy in the preparation and delivery of a health education session
Attendance	5	Punctuality, consistency, and engagement during sessions
	50	

Online assessment

The students were assessed for CPSCR and epidemiological exercise by means of online presentations from their respective places (hostels or hometowns), while all the faculty members (5-7 per group) evaluated from their workplace. 

The end posting assessment was conducted as a multiple-choice question (MCQ)-based test in online mode.

For each batch, three sets of comparable question papers covering all domains were prepared in Google Forms (Google LLC, Menlo Park, California, United States). Each set consisted of 15 questions, with one question in each section. Separate marks were allotted to each section to avoid ambiguity in marking. Pre-testing of questions was done with resident doctors for content and timing. 

The first section of the form had general instructions like how to navigate the forms and the marking scheme, as well as the time duration of the assessment. 

The students were then given a six-digit password (which was common for all three sets). This marked the onset of the assessment, and a stopwatch was used to note this timing. Due to issues with bandwidth for most of the students, they could not be asked to switch on their webcams.

Throughout the conduct of the test, real-time troubleshooting and support were provided to the students by a preceptor to troubleshoot the problems related to difficulty in logging in, opening the form, performing the test, or submitting it. 

Student feedback 

On the same day after the completion of the online assessment, feedback was obtained from the students. A Google Form was administered, which had both closed- and open-ended questions. The closed-ended questions had multiple-choice responses. The questions were designed to capture the usability, functionality, and design of the online teaching. The feedback questionnaire was adapted from published literature, pilot-tested with resident doctors, and refined for clarity. Although formal validation was not done, content relevance was ensured by faculty consensus. Two faculty members independently coded open-ended responses; disagreements were resolved by discussion, and inter-rater agreement was checked qualitatively. For the open-ended questions, students were asked for their comments and suggestions. Based on the available literature, we tried to evaluate the online teaching and assessment in terms of its usability, functionality, and design [6-8].

Data analysis

The responses were analyzed in Microsoft Excel (Microsoft Corporation, Redmond, Washington, United States), and the results were expressed as percentages in various domains.

The open-ended responses were systematically analyzed using a thematic analysis approach. All the responses were read multiple times, and meaningful units of text were identified, coded manually, and grouped into categories based on recurrent ideas. Codes were then collated into sub-themes and broader themes through an iterative process of comparison and refinement. Student quotations were used to illustrate key themes and to ensure the authenticity of interpretation. Themes related to online assessment were also derived, capturing student views on the adequacy of instructions, content coverage, identification of learning gaps, time constraints, technical barriers, design issues, and opportunities for academic misconduct (cheating during online assessment). The open-ended feedback was compiled, and themes were identified, providing a clearer, though brief, description of the qualitative analysis process.

Ethical considerations

The participation in the study was voluntary. Informed consent was obtained online through Google Forms. Identification details of the students were delinked from the data before analysis. Anonymity of the responses and confidentiality of the data were ensured. The feedback provided by the students did not have any impact on the assessment. 

## Results

A total of 58 students responded to the feedback. The majority (n=48; 85%) of the students were satisfied with the online adaptation of the UHP. They reported that interaction and teamwork were possible in the given online adaptation. The students found it convenient, time-efficient, and less stressful. However, access to the internet was a major challenge. Two students (3.4%) reported that data charges were very high. Poor network was another common problem. 

A recurring theme was the inability of virtual sessions to replicate the richness of in-person community-based learning, with students emphasizing the unique value of physical presence and direct observation: 

"Nothing feels like physical presence. Virtual feels very different."

"Physical mode would make us more exposed to the things as we see with our own eyes."

Despite these limitations, many students acknowledged that online teaching was the only feasible alternative during the COVID-19 pandemic, recognizing the necessity of streamlining digital platforms under such circumstances:

"With COVID spreading at the rate it is, we have to streamline online functions more."

"Online is the only option in the present situation."

As far as assessment is concerned, the majority, i.e., 46 (79.3%), of the students were satisfied with the instructions given to them about the online assessment. While 38 (66%) students felt that all the topics taught during the posting were adequately covered in the assessment, 42 (72.4%) opined that the assessment had helped them to identify the areas that needed attention. Regarding the time allotted for attempting the test, 32 (53%) students felt that it was less than required. Nearly half of the students (n=29) reported that they faced a problem in the online assessment, and 26 (45%) did not report any problem, while three (5%) did not respond to this question.

Common problems or challenges

The common problems or challenges faced were as follows:

Time

The most commonly cited problem was inadequate time allotted for the test. Most of the students felt that the time for responding to the questions was appropriate; however, shifting from one question to another was time-consuming.

Internet Accessibility

Since the students attempted the assessment from their respective hometowns, a few students reported having limited access to the internet. 

Design and Layout of the Assessment

There were a few issues, such as difficulty in going back to a previous question and shifting from one question to another. 

Ease of Cheating

The majority (66%) of the students felt that there was a scope for cheating in online assessments, and 49 (84%) of them admitted that they attempted to cheat. 

**Figure 1:**
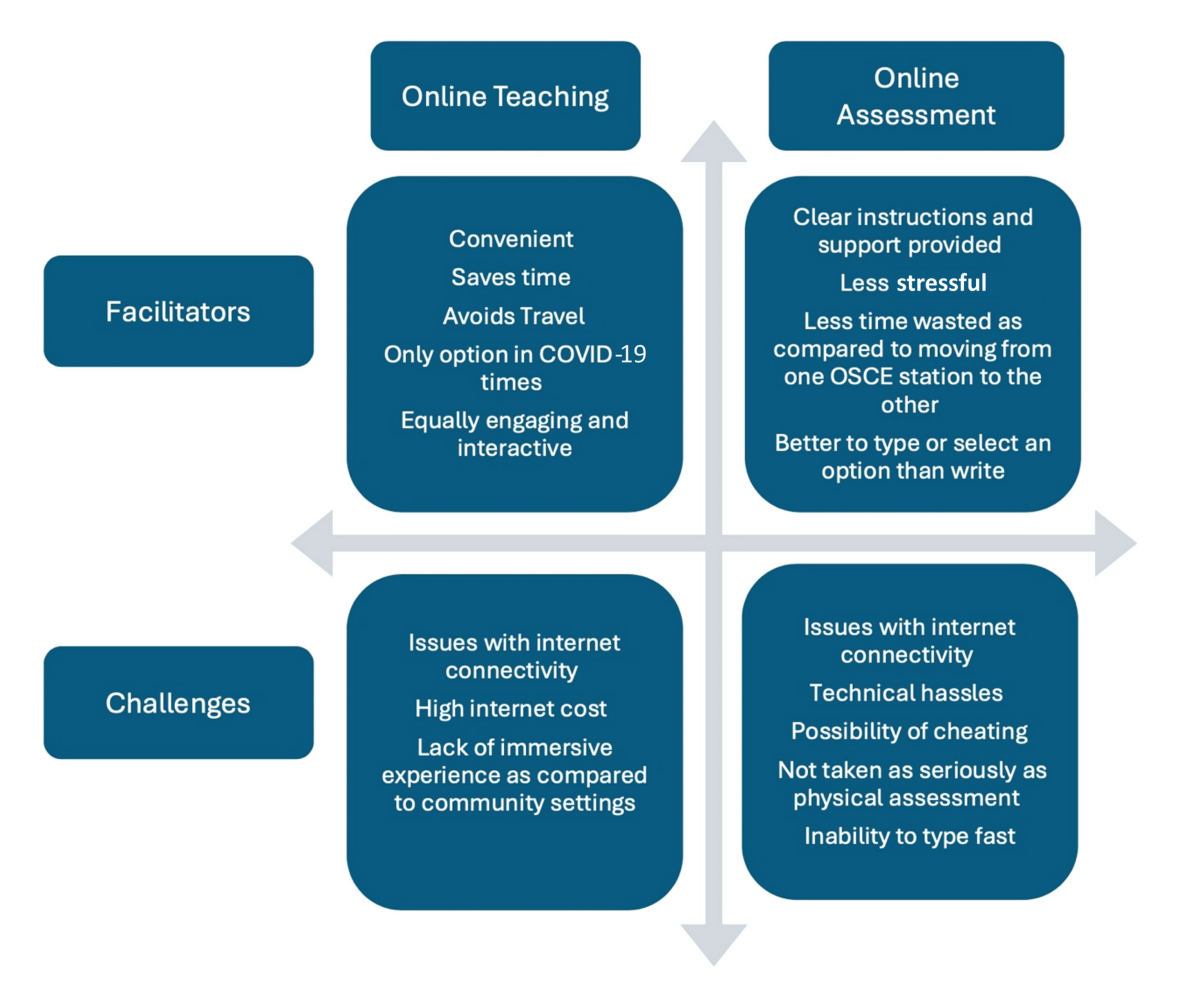
Facilitators and challenges faced by medical students in the online adaptation of the UHP OSCE: Objective Structured Clinical Examination; UHP: Urban Health Posting

Figure 1 summarizes the facilitators and challenges faced by the students in the online adaptation of the UHP. 

Although the students reported some issues, they appreciated the support and instructions provided. In response to the question that asked their preference for classroom vs. online assessment in the future, 31 (53%) chose classroom, while 27 (47%) preferred online assessment. Students acknowledged the disadvantage in online tests due to their typing skills, though it was perceived to be less stressful than classroom tests.

For improving the assessment in the future, the students suggested a user-friendly layout, with easy navigation, and switching on the webcams to prevent cheating. A few of them felt that viva voce in video conference mode was better than multiple-choice or image-based questions.

## Discussion

In light of the COVID-19 pandemic and the resulting lockdown in 2020, medical education at our institute underwent significant restructuring. The UHP that traditionally relied heavily on field-based, experiential learning was adapted to an online mode. To maintain interactive and participatory learning, each didactic session included time for student engagement. 

One of the key challenges in transitioning to an online mode was the adaptation of the CPSCR, a central activity in the UHP. The presentation in group sessions and detailed feedback on content, analysis, and communication skills preserved the holistic patient review model while adhering to lockdown restrictions.

The online adaptation of the community research component also culminated in group presentations to the faculty, simulating a scientific conference environment and fostering research communication skills.

The nearby health facility visit enabled the experiential learning within a constrained environment, encouraged contextual understanding of health services, and fostered critical thinking. Despite the absence of centralized field postings and visits to public health facilities, the module, within the given constraints, successfully simulated real-world exposure to a considerable extent using a decentralized, student-centered approach.

The students acknowledged certain benefits of online CBT, including ease of access, time efficiency, and reduced logistical constraints. Similar findings were reported by Venugopal and Dongre [9]. Research by Dutta et al. highlighted that students valued the opportunity to learn through online platforms [10]. 

However, the students in our study emphatically highlighted the inherent limitations of online adaptation in replicating the richness of in-person CBT. While e-learning provides flexibility and continuity, it lacks the sensory and contextual immersion essential for holistic learning in community settings.

Rafi et al. also reported that while students recognized the flexibility of online platforms, they expressed dissatisfaction with the lack of hands-on community engagement and limited interaction with faculty and peers, which are integral to community-based learning [11]. Furthermore, the importance of real-time feedback was highlighted in our study and emphasized by Iahad et al., where students emphasized the need for structured, timely feedback to support learning in the virtual environment [12].

Our paper also highlights common issues and challenges faced while conducting online assessments. The most common challenge was internet connectivity and a lack of computer skills, including typing. Other studies have also identified similar challenges [13,14].

In our study, it was foreseen that cheating could be possible. The same has been identified as an important disadvantage of online assessment in past studies by Mellar et al. and Kocdar et al. [15,16]. We tried to overcome this by having different sets of question papers, having a different sequence of questions in similar sets, and providing just adequate time to answer. A response validation was built in so that each student would attempt the question paper allotted to him/her. Switching on the webcam while doing the test was another option; however, there was a limitation of bandwidth. Google Forms were used, which had their own limitations.

Other limitations of this study included the use of self-reported data collection and the potential for response bias from students, though the open-ended feedback forms were collected anonymously to limit the possibility of response bias. In addition to self-reported data and response bias, this study was limited by its small, single-institution scope, a lack of formally validated feedback tools, and the absence of comparison with traditional CBT outcomes. These factors restrict the generalizability of findings beyond similar contexts. Moreover, as the study only captured student perceptions at one point in time, causal inferences cannot be made. Findings should be interpreted within this specific context.

However, the students appreciated the support and troubleshooting provided to them throughout the conduct of the assessment and the adequate instructions provided to them before the assessment. On the contrary, non-availability of technical support has been reported to be a problem in some other studies [12,17].

The students in our study suggested that detailed feedback regarding their performance in the test should have been provided. A similar suggestion was given by students in the study conducted by Iahad et al. [12].

The advantages of online assessment reported in our study, such as time-saving, ease, and flexibility, have been reported in studies conducted earlier [10,11].

The present study highlights critical considerations in the design and implementation of online adaptations for CBT and assessment in undergraduate medical education. While there is no substitute for CBT, its online adaptation was the only option available at the time of the COVID-19 lockdown. Key factors identified include the importance of aligning assessments with learning objectives, ensuring user-friendly interfaces, and ensuring student engagement. The present study also identifies key factors of evaluation that should be taken into account while developing online tests, which may help to design better assessment systems. 

## Conclusions

The online adaptation of the UHP was perceived to be convenient and engaging, with students reporting that interaction and teamwork were still possible. However, field-based experiential learning was missed. The study's descriptive design, while providing valuable insights into student perceptions, does not allow for a definitive comparison of learning effectiveness between online and in-person formats. Future research should consider a comparative study to objectively evaluate learning outcomes and skill acquisition in both online and traditional CBT settings.
